# Dual Growth Factor Release From Collagen Based Multilayer Films on Ti‐Surfaces Enhances Periimplant Bone Formation and Angiogenic Activity—An Experimental In‐Vivo Study

**DOI:** 10.1111/clr.70045

**Published:** 2025-09-12

**Authors:** Philipp Kauffmann, Susanne Wolfer, Christina Behrens, Pauline Schlosser, Christian Dullin, Uwe Schirmer, Klaus Liefeith, Henning Schliephake

**Affiliations:** ^1^ Department of Oral and Maxillofacial Surgery George‐Augusta‐University Göttingen Germany; ^2^ Department of Diagnostic and Interventional Radiology George‐Augusta‐University Göttingen Germany; ^3^ Department of Diagnostic and Interventional Radiology University Hospital Heidelberg Heidelberg Germany; ^4^ Max Plank Institute for Multidisciplinary Sciences Göttingen Germany; ^5^ Institute for Bioprocessing and Analytical Measurement Techniques Heiligenstadt Germany

**Keywords:** angiogenesis, bone formation, bone morphogenic proteins, collagen, heparin, layer‐by‐layer (LbL), polyelectrolyte multilayer, vascular endothelial growth factor

## Abstract

**Objectives:**

The aim of the present study was to assess the effect of dual growth factor release from collagen‐based polyelectrolyte multilayer films (PEMs) on titanium implants on periimplant bone formation and angiogenetic activity in minipig mandibles.

**Material and Methods:**

Disc shaped Ti implants (5 × 7 × 1 mm) were coated with polyelectrolyte multilayer films and loaded with rhBMP‐2, rhVEGF165, and a combination of both. Uncoated and unloaded Ti implants served as controls. The implants were inserted press‐fit into 5 mm trephine cavities in minipig mandibles and evaluated after 4 and 13 weeks for bone formation, bone implant contact, and periimplant expression of CD31.

**Results:**

After 4 weeks, there was no significant difference in periimplant bone formation nor bone‐implant contact between the different surface conditions. CD31 expression was significantly increased around PEM coated implants loaded with VEGF and BMP each. After 13 weeks, bone implant contact and bone formation were significantly increased only around PEM coated implants with simultaneous release of BMP‐2 and VEGF165. Expression of CD31 was significantly enhanced around all PEM coated implants loaded with growth factors regardless of the type or combination.

**Conclusion:**

It is concluded that simultaneous release of rhBMP‐2 and rhVEGF165 may be required to achieve significant improvement of periimplant bone formation and bone implant contact. Angiogenesis as reflected by CD31 expression is enhanced by both rhVEGF165 and rhBMP‐2 to a similar extent without an additive effect of simultaneous release.

## 1 Introduction

Titanium implants have proven to provide a reliable means of anchorage for dental prostheses. However, local and systemic conditions such as radiation therapy or osteoporosis can lead to deterioration of structural and biological bone quality and jeopardize the implant success rate in individual patients (Keller et al. 2004; Temmerman et al. 2019; Lemos et al. 2023; Schiegnitz et al. 2022). Numerous attempts have been made to enhance the biological activity of dental implant surfaces in order to improve periimplant bone formation and increase the degree of osseointegration. These included modification of surface roughness and nano‐topography, biomimetic coating and nano‐coating with anorganic material such as Calcium Phosphates as well as coating with organic molecules such as bisphosphonates, RGD peptides, collagen, and polypeptide growth factors (for review see Souza et al. 2019; Kunrath et al. 2024; Sarvaiya et al. 2025).

The strategy of modifying biomaterials and implant surfaces for the delivery of osteogenic signals has been pursued in many ways. Among these approaches, adsorptive dip coating with milligrams of growth factor (BMP in particular) has been clinically applied, which has resulted in rapid release of large amounts of growth factor raising safety concerns due to untoward effects such as swelling and implant loosening in clinical and preclinical settings (e.g., Burkus et al. 2002; Leknes et al. 2008; Fiorellini et al. 2005). More elaborate methods of targeted binding and delivery of growth factors have employed monolayer coatings of implant surfaces using anchoring molecules on the one hand and multilayer coatings of multiple layers of polyelectrolyte molecules on the other. While monolayer coatings using oligonucleotides, dopamine, or chitosan have been able to bind and release growth factor amounts in the ng range (Schliephake et al. 2012; Shi et al. 2009; Huang et al. 2022), the layer‐by‐layer coatings have shown to accommodate amounts of growth factor in the μg range (Guillot et al. 2013; Gilde et al. 2016; Bouyer et al. 2016; Gronowicz et al. 2017; Jacobs et al. 2017; Zhang et al. 2018; Damanik et al. 2019). For binding and release of polypeptide growth factors, heparin, a naturally occurring glucosaminoglycan, has been frequently used as anionic polyelectrolyte in polyelectrolyte multilayer coatings (PEMs) because it provides binding sites for a number of bone growth factors such as bone morphogenic proteins (BMPs) and vascular endothelial growth factor (VEGF) (Ishihara et al. 2019; Wigmosta et al. 2021; Ludolph et al. 2022; Behrens, Kauffmann, von Hahn, Giesecke, et al. 2022; Behrens, Kauffmann, von Hahn, Schirmer, et al. 2022; Lu et al. 2023).

As tissue integration of an implant is not only promoted through the release of biologically active growth factors but also by surface characteristics that enhance cell attachment and proliferation on the implant surface, a combination of an osteoconductive surface characteristic and osteoinductive activity through the delivery of growth factors could be even more promotive for peri‐implant bone formation and osseointegration. Collagen, as the major organic component of bone tissue, provides numerous binding sites for cellular attachment (Hynes 2002) and has been shown to significantly promote osteogenic differentiation of mesenchymal stem cells exposed to collagen‐based nanofilms (Hwang et al. 2019). Moreover, nanoanchored collagen coating on implant surfaces has significantly increased bone‐implant contact rates in vivo (Schliephake, Scharnweber, et al. 2005). As a cationic counter‐polyelectrolyte for heparin, collagen is thus an attractive component for the construction of polyelectrolyte multilayer films (PEMs) designed for the enhancement of bone formation (Ao et al. 2013; Ferreira et al. 2016; Brito Barrera et al. 2020; Liu et al. 2021).

As bone formation involves a delicately orchestrated sequence of signaling molecules, the incorporation of more than one osteogenic growth factor into drug‐releasing appliances has been requested (Bayer et al. 2015; Zhang et al. 2025). Earlier approaches to multiple growth factor loading of implants that have used dip coating of Calcium Phosphate coated implant surfaces with BMP2 and VEGF165 have not been able to show significantly enhanced periimplant osteogenic differentiation and bone formation (Ramazanoglu et al. 2011, 2013). Little is known so far about the effect of dual growth factors loaded to and released from collagen‐based PEM films on in vivo osteogenesis and angiogenesis (Liu et al. 2021). It was thus the aim of the present study to establish a collagen and heparin‐based polyelectrolyte multilayer coating on titanium implants loaded with two growth factors involved in osteogenesis (rhVEGF165 and rhBMP‐2) and to assess the individual contribution of these signaling molecules on periimplant angiogenic activity and bone formation in conjunction with the Col‐Hep PEM films.

The current study on the combined release of BMP and VEGF from collagen‐based multilayer coating of implant surfaces was conducted as part of a series of experiments both in vitro and in vivo together with experiments evaluating poly‐L‐Lysin‐Heparin (PLL‐Hep) multilayer coatings. Both multilayer concepts had been thoroughly assessed in vitro (Behrens, Kauffmann, von Hahn, Giesecke, et al. 2022; Behrens, Kauffmann, von Hahn, Schirmer, et al. 2022; Schliephake and Liefeith 2022; Ludolph et al. 2022) and have now been evaluated in vivo. The PLL‐Hep study had in focus different timing patterns of growth factor release of BMP and VEGF (simultaneous vs. sequential, single vs. dual) from different PLL‐HEP multilayer architectures (Kauffmann et al. 2025), whereas the current study looked at simultaneous release of BMP and VEGF and the effect of using collagen that is known to increase cell attachment and osteoconductive bone growth. Both studies were performed together in an in vivo experiment in order to follow the 3R principles by sharing uncoated and unloaded control implants and by sharing the pig mandible as the anatomical site of insertion.

## 2 Materials and Methods

### 2.1 Specimen Fabrication

Commercially pure sandblasted titanium discs (7 × 5 × 1 mm, KLS Martin, Tuttlingen, Germany) were used as carriers for the PEM films for in vitro and in vivo evaluation. Before coating, the samples were etched in 5.1 M hydrochloric acid and 4.6 M sulfuric acid solution for 300 s at 108°C, as previously described (Scharnweber et al. 2010).

### 2.2 Collagen Multilayer Coating of Ti‐Discs

The Ti‐specimens were coated with a collagen‐heparin based polyelectrolyte multilayer film (Col‐Hep‐PEM) for growth factor loading later on. Poly‐L‐lysine (PLL, 30–70 kDa, Sigma Aldrich, Taufkirchen, Germany) and heparin (Hep, 50 mg/mL, from porcine intestinal mucosa) were assembled as an initial double layer on the metal surface. Subsequently, nine double layers of collagen I (rat tail collagen type I, ibidi, Gräfeling, Germany, 5 mg/mL) and heparin were added to the surface, resulting in a (PLL‐Hep1) (Col‐Hep9) polyelectrolyte multilayer film, referred to as Col‐Hep PEM. The polyelectrolytes were dissolved in 5 mM acetate at a concentration of 1 mg/mL. Film construction was performed semi‐automatically employing a dipping robot (DR3, Riegler & Kirstein, Germany). The cleaned Ti specimens were first soaked in the PLL solution for 5 min, followed by three washing steps in deionized water to remove unbound PLL. Subsequently, heparin adsorption was performed in an identical fashion. For the Col‐Hep film construction, the PLL solution was replaced by a collagen solution (1 mg/mL collagen I) and the dipping protocol was modified by increasing the dipping time to 30 min with 3 subsequent washing steps for both collagen and heparin. The Col‐Hep dipping cycles were performed nine times with final rinsing in deionized water and subsequent air drying.

#### 2.2.1 In Vitro Experiments

##### 2.2.1.1 Growth Factor Loading

The PEM coated specimens and uncoated Ti specimens were loaded with rhBMP‐2, rhVEGF165, and a combination of both growth factors (see below). For in vitro release testing, the titanium discs were placed into 3D‐printed silicone mounts in groups of three discs, exposing only one side of the disc. They were dipped overnight in 160 μL of a loading solution with either rhBMP‐2 (75 μg/mL; Chinese Hamster Ovary cell‐derived, PeproTech, Hamburg, Germany) or rhVEGF165 (75 μg/mL; Human Embryonic Kidney 293 cell‐derived, ThermoFischer, GIBCO, Darmstadt, Germany), corresponding to 3 μg per specimen. The discs with dual growth factor loading were first incubated with rhVEGF165, followed by rhBMP‐2. The concentrations of growth factors in the loading solutions for rhBMP‐2 and rhVEGF165 had been defined during previous experiments (Behrens, Kauffmann, von Hahn, Giesecke, et al. 2022). Subsequently, the supernatant was stored in a reaction tube for further use, and the loaded specimens were washed twice with deionized water and shortly air‐dried at RT.

For the in vivo experiments, the PEM coated and uncoated titanium discs were individually loaded with growth factors on both sides. The specimens were placed into a single well each with 3D‐printed silicone containers and incubated overnight in 80 μL of loading solutions with rhBMP‐2 (75 μg/mL; Chinese Hamster Ovary cell‐derived, PeproTech, Hamburg, Germany) or rhVEGF165 (75 μg/mL; Human Embryonic Kidney 293 cell‐derived, ThermoFischer, GIBCO, Darmstadt, Germany), corresponding to 6 μg growth factor per specimen reservoir. The samples with dual growth factor loading were first incubated with rhVEGF165 followed by rhBMP‐2. Like the specimens for the in vitro experiments, the supernatant was stored, and the specimens were washed twice with deionized water and stored at 4°C after being air‐dried at RT.

Eight surface conditions of the Ti specimens were prepared:

- Col‐Hep PEM loaded with rhBMP‐2
- Col‐Hep PEM loaded with rhVEGF165
- Col‐Hep PEM loaded with rhBMP‐2 and rhVEGF165
- Col‐Hep PEM without growth factor loading (Control 1)
- Uncoated Ti surface loaded with rhBMP‐2 (Control 2)
- Uncoated Ti surface loaded with rhVEGF165 (Control 3)
- Uncoated Ti surface loaded with rhBMP‐2 and rhVEGF165 (Control 4)
- Uncoated Ti surface without growth factor loading (Control 5)

Loading efficacy for rhBMP‐2 and rhVEGF165 was assessed indirectly by measuring the remaining amount of both growth factors in the stored supernatant of the coating procedure. A Bicinchoninic Acid (BCA) Protein Assay Kit (ThermoScientific, Darmstadt, Germany) with bovine serum albumin (BSA) as standard was applied using 25 μL of each standard (working range 25–2000 or 5–250 μg/mL). The samples were replicated into a microplate well (96‐well plates). After addition of 200 μL of working solution to each well, the plates were placed on a plate shaker for 30 s (37°C, 400 rpm, THERMOstar, BMG LABTECH, Ortenberg, Germany) and incubated at 37°C for 30 min. Absorbance at 562 nm was measured with an ELISA plate reader (SpectraMax M2, Molecular Devices, San Jose, CA, USA) at room temperature (RT).

##### 2.2.1.2 Release Experiments

The loaded Ti specimens with/without PEM coating were incubated in 250 μL DMEM in 24‐well plates supplemented with 2% FCS and 1% penicillin/streptomycin at 37°C in a 5% CO_2_ atmosphere at 70 rpm (Celltron, InforsHT, Einsbach, Germany). The medium was collected and replaced after 24, 48, and 72 h and every 3 days thereafter until Day 21. For stabilization of the supernatants, a protease inhibitor was added (ROCHE Diagnostics, Mannheim, Germany). The released amounts of rhBMP‐2 and rhVEGF165 were assessed using a Human/Murine/Rat BMP‐2 and Human VEGF TMB ELISA Development Kit (PeproTech, Hamburg, Germany), according to the instructions of the manufacturer. Recombinant human hBMP‐2 (PeproTech) and rhVEGF‐165 (ThermoFisher, Gibco) were used as standards. All in vitro evaluations were performed twice on three specimens each.

### 2.3 In Vivo Experiments

#### 2.3.1 Sample Size Calculation

The eight different surface conditions were scheduled for evaluation after 4 and 13 weeks each. The experiments were combined with the testing of 6 other PEM formulations, the results of which will be reported elsewhere (Kauffmann et al. 2025). Following the 3R principles, the results obtained for the Ti controls were shared between the two experiments. For sample size calculation, a Family‐Wise‐Error‐Rate of 5% across the 14 comparisons per interval was intended to be maintained, resulting in *α* = 5%/14 = 0.36%. The power level was set to 80%. A difference in mean values between the surface conditions of 10% was assumed, and a standard deviation of *σ* = 10% within each group. A z‐approximation was used to calculate the sample size based on these figures, resulting in a number of 6 animals required per interval.

#### 2.3.2 Surgical Procedures and Animal Care

The mini pig mandible was chosen as a model for the experiments as the bone biology in this species compares well with human biology (Pearce et al. 2007). The unit of evaluation was the individual animal. Twelve female animals were used (age: 2–3 years, weight 44.6 ± 7.6 kg) and randomly allocated to the intervals of evaluation of 4 weeks and 13 weeks, respectively, resulting in two groups of six animals. Randomization was generated by drawing lots. All surgical procedures, housing, and animal care were carried out in accordance with the German legislation for animal protection and the regulations for animal experiments of the state of Lower Saxony. The trials were reported and admitted under the license number 20/3554 of the Office for consumer protection and food safety of Lower Saxony (LAVES). Animals were held in groups of 2–3 animals in cages with concrete floors with sawdust bedding and wooden walls. They were allowed to accommodate for 4 weeks prior to the beginning of the clinical procedures. All animals presented in good health. All surgical procedures were conducted in the animal facilities of the University Medicine Goettingen following the ARRIVE guidelines (Kilkenny et al. 2012). The experiments were conducted between 02/2022 and 06/2022. Qualified veterinarians performed the sedation and general anesthesia as well as the postoperative care. Sedation was initiated by an orally administered dose of 0.5 mg/kg body weight of Diazepam followed by intramuscular injection of 10 mg/kg body weight Ketamine and 2 mg/kg body weight Azaperone after approximately 20 min. General anesthesia was induced with titrated i.v. administration of Thiopental followed by endotracheal intubation. Anesthesia was maintained using 2% to 4% of Isoflurane, supported by Piritramid and Ketamine to add analgesic capacity. A Dexpanthenole lotion (Bepanthen, Bayer AG, 51368 Leverkusen, Germany) was used to cover the eyes.

**FIGURE 1:**
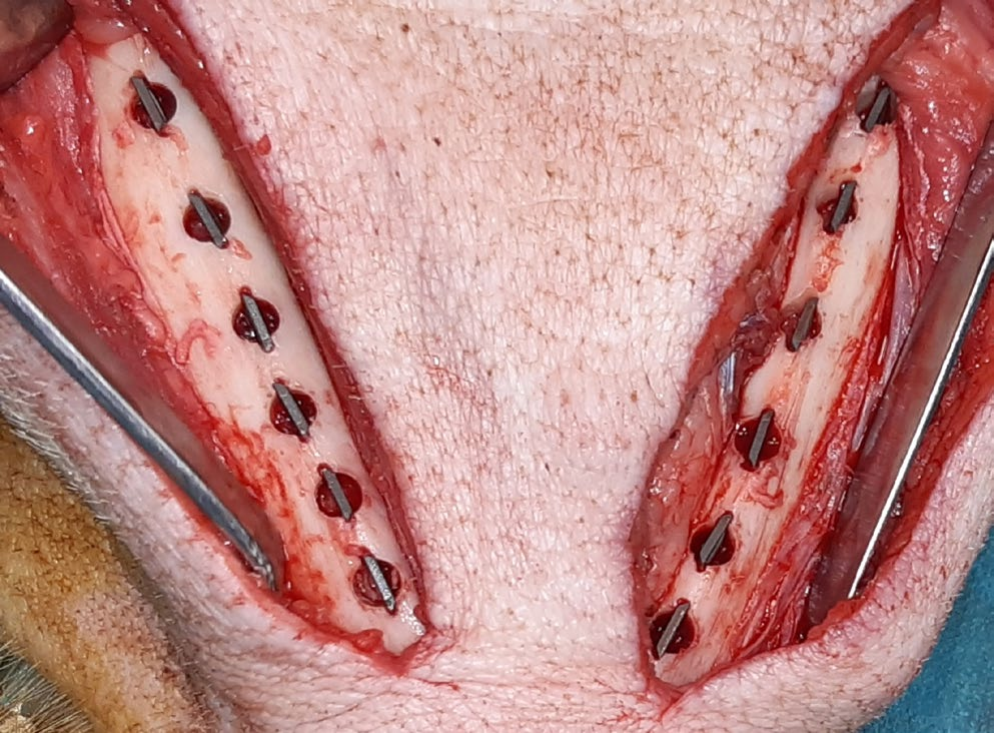
Clinical picture of implant insertion into the lower border of a minipig mandible.

As mentioned above, the experiments in the present study were combined with experiments from a different study using PLL‐Hep coated implants (Kauffmann et al. 2025). For both studies, all in all 14 cylindrical cavities of 5 mm diameter and 5 mm depth were created in the lower border of the mandible through an extraoral approach. Vertical bone cuts of 0.5 mm depth were created at the mesial and distal sides of each defect using a fissure burr to allow for press fit insertion of the 7 mm broad specimens into the trephine cavities (Figure 1). In the present study, five of the 14 cavities were used for the insertion of the 5 control implants and 3 cavities were used to insert the growth factor loaded implants (see Section 2.2.1.1). The different surface conditions were randomly assigned to the defect locations along the mandibular border by drawing lots. After insertion, the periosteum overlying the cavities was excised to exclude bias in bone formation due to excessive periosteal bone regeneration. Wound closure was performed in layers using resorbable sutures (Vicryl 3.0, Ethicon, Norderstedt).

During the immediate postoperative period (1 week) of both series of operations, animals were visited twice per day. For reduction of postoperative pain, 0.6 mg Buprenorphin was administered intravenously twice per day combined with 5 mg/kg body weight Carprofen during the first 3 days. If animals showed signs of discomfort 5–7.5 mg/kg body weight Carprofen was administered orally. After a maximum of 5 days, all animals were well.

### 2.4 Histologic Preparation and Morphometry

The mandibles of six animals each were removed after 4 weeks and 13 weeks, with retrieval of the Titanium implants together with adjacent bone using a diamond saw (EXAKT, Robert‐Koch‐Str. 5, 22851 Norderstedt, www.exakt.de). Specimens were dehydrated and embedded into Technovit 9100 (Heraeus Kulzer GmbH, Philipp‐Reis‐Str. 8/13, 61273 Wehrheim, Germany). Between 10 and 12 thick‐section specimens (Donath and Breuner 1982) were produced from each implant and its surrounding bone perpendicular to the axis of the trephine cavity. Four of the resulting specimens were surface stained with Alzarine‐Red/Methylene Blue. For histomorphometry, two areas of interest were defined: (i) the whole area of the trephine defect and (ii) a 300 μm thick area adjacent to the implant surface. This periimplant zone was subdivided into three layers of 100 μm thickness each on both sides of the implant cross section, creating an (i) immediate, (ii) intermediate, and (iii) remote tissue layer in relation to the implant surface.

For morphometric evaluation, specimens were scanned using a digital scanning device (Dotslide‐System2.0, Olympus Deutschland GmbH, Wendenstraße 14–18, 20097 Hamburg, Germany). The resulting digital image data were analyzed using a custom‐made Python3‐based image analysis pipeline utilizing the common Python modules scikit‐image, matplotlib, opencv, and pandas.

**FIGURE 2:**
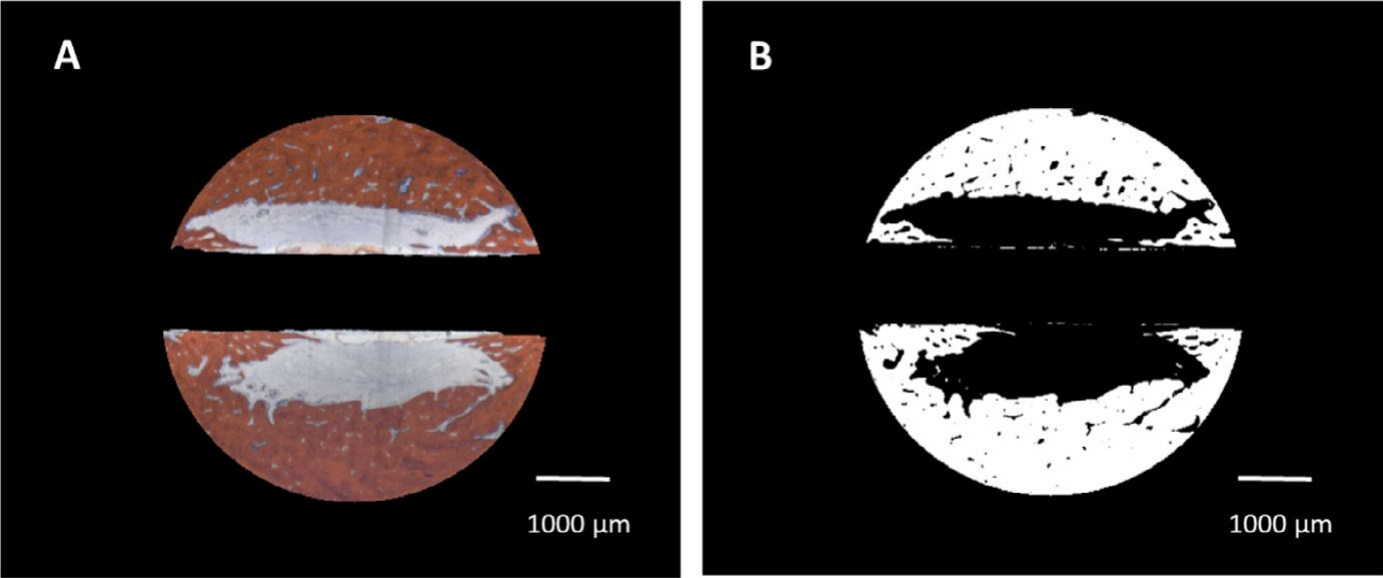
(A) Definition of the trephine defect for morphometric evaluation, Bar: 1000 μm. (B) Digitization of the bone area. Bar: 1000 μm.

Primary outcome parameters were as follows:

- Bone area/bone density: the algorithm automatically identified the color of the Alizarine Red stained areas in the cross‐section specimens and assessed the area occupied by bone both in absolute values (bone formation [BF]) and in relation to each section area (bone density [BD]) by pixel counting. Pixels were converted to mm^2^ using the calculated pixel size of 17.43 μm^2^/pixel (Figure 2A,B). Bone density was only evaluated for the trephine defects as a whole. To account for variations in the appearance of the color of Alizarine Red in the different cases and in cases in which the new formed bone covered the entire trephine defect, parameters were manually adjusted.
- Bone implant contact (BIC). The algorithm identified the surface area occupied by bone by image analysis routines and calculated the bone‐implant contact (BIC) as a percentage of the occupied surface area. In brief, the surface of the identified cross‐section of the implant was enlarged by 1 pixel (approx. 4.18 μm) and limited to the trephine defect size. The resulting mask was multiplied with the bone mask, and the ratio of those pixels to the entire surface of the implant was calculated.

Measurements were performed by one blinded examiner, who was calibrated during introduction to the image analysis system. Outcome parameters were assessed individually for each cross‐section; mean values were calculated for each disc from 3 to 4 cross‐sections.

### 2.5 Immunohistochemical Preparation and Evaluation of Immunofluorescence

Technovit embedded thick‐section specimens (see Section 2.4) were mounted on glass slides (Paul Marienfeld GmbH, Lauda‐Koenigshofen, Germany). After preparation of tissue sections with a resulting thickness of 70–100 μm the sections were incubated three times with xylene, 20 min each, and placed three times (twice for 20 min and once overnight) in MEA (2‐methoxyethylacetate, Merck, Darmstadt, Germany). The specimens were then rehydrated in descending concentrations of ethanol (100%, 96%, 70%, twice for 2.5 min each) and washed twice in deionized water for 2.5 min each.

For the immunofluorescence staining, the deplasticized and rehydrated bone tissue sections were incubated in 1× citrate‐based TR buffer, pH 6.0 (Target Retrieval Solution, Agilent Dako, Waldbronn, Germany) for 30 s at 121°C, followed by incubation for 10 s at 90°C using a standard pressure cooker (PASCAL S2800, Dako, Hamburg). Subsequently, the sections were incubated for 5 min at room temperature (RT), washed for 10 min in deionized water, and lastly washed three times in PBS for 5 min each. Next, the samples were incubated for 1 h at RT in blocking buffer (10% goat Serum Block in PBS, Histoprime Biozol, Eching, Germany). For the detection of CD31, the specimens were incubated with an anti‐CD31 antibody (CD31/PECAM1, Platelet/endothelial cell adhesion molecule‐1, ABIN 726140, 1:100; antikoerper‐online.de, Aachen, Germany) at 4°C overnight. After washing three times in PBS, 5 min each, the specimens were labeled using the secondary antibody Alexa Fluor 647 (ab 150079, 1:500; Abcam, Cambridge, UK) by incubation for 1 h at RT, followed by washing three times in PBS for 5 min each. Subsequently, nuclei were counterstained with DAPI (1:1000; Sigma‐Aldrich Merck, Darmstadt, Germany) for 10 min at RT. Finally, the sections were washed in PBS (three times, 5 min each) and mounted with Fluor Save Reagent (30 min at RT and overnight at 4°C; Merck Millipore, Darmstadt, Germany).

The antibodies and DAPI were diluted using Antibody Diluent (Agilent Dako, Waldbrunn, Germany). All incubations, including blocking, were performed in a humidity chamber.

**FIGURE 3:**
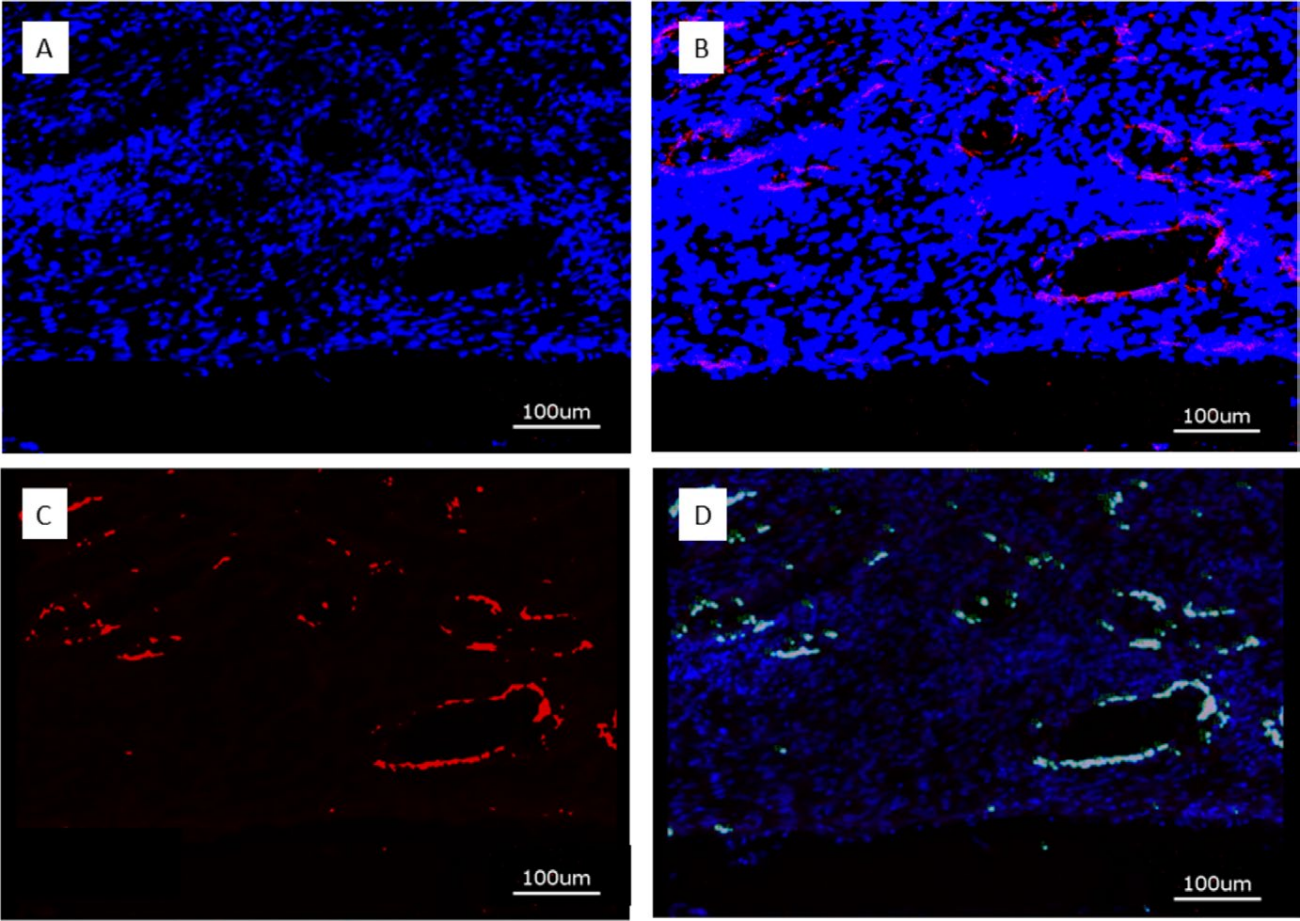
(A) DAPI stain of nuclei in periimplant tissue (blue); (B) Overlay of DAPI stain and CD31 expression (red); (C) Isolation of CD31 positive area; (D) Digitization of CD31 positive area, Bar 100 μm.

After immunostaining, the specimens were analyzed with the KEYENCE BZ‐X710 microscope (Keyence, Neu Isenburg, Germany) using a Cy5 filter (OP‐87766) for detection of the target protein CD31 and the DAPI filter (OP‐87762). Sections stained without the primary antibody served as controls. One cross‐sectional specimen of each surface condition per animal was evaluated. Quantitative analysis was performed on digital images of the specimens at 20‐fold magnification corresponding to an image size of 725 × 543 μm displaying the implant surface at the lower border of the field of view and periimplant tissue up to a distance of approximately 400 μm thickness. Thirteen images, placed on the upper (7) and lower (6) edge of the cross sections, were analyzed. Quantification of CD31 expression was measured in μm^2^ using the BZ‐X‐Analyzer tool filter for red light; overlay images with DAPI stained nuclei were used for analysis (Figure 3: A through D).

### 2.6 Statistics

Data are presented as means ± standard deviation (SD). Friedman tests (SPSS Statistics 26.0, http://support.spss.com) were used to compare bone formation (BF), bone density (BD) and bone‐implant contact (BIC) as well as expression of CD31 between the experimental surfaces. Additional Wilcoxon tests were used for pairwise comparison. All tests were performed at a significance level of *p* < 0.05.

## 3 Results

### 3.1 In Vitro Experiments

#### 3.1.1 Growth Factor Loading

Col‐Hep‐PEM coated samples loaded with rhBMP‐2 and rhVEGF165 incorporated a mean amount of 4.30 μg/cm^2^ rhBMP‐2 (SD 0.77) and 4.02 μg/cm^2^ rhVEGF165 (SD 0.43) (Table 1). Col‐Hep PEMs loaded with rhVEGF165 picked up 4.48 μg/cm^2^ (SD 0.67) on average, and Col‐Hep PEMs loading with rhBMP‐2 only were loaded with a mean amount of 5.69 μg/cm^2^ growth factor (SD 1.08). Loading of the bare Titanium surfaces of the uncoated specimens with both growth factors adsorbed 2.56 μg/cm^2^ rhBMP‐2 and 4.57 μg/cm^2^ rhVEGF165. Loading with only one growth factor accommodated average amounts of 4.37 μg/cm^2^ rhVEGF165 and 2.26 μg/cm^2^ rhBMP‐2, respectively.

**TABLE 1:** Growth factor loading (rhBMP2/rhVEGF_165_).

	rhBMP2 (μg/cm^2^)		rhVEGF_165_ (μg/cm^2^)	
Col‐Hep/VEGF+BMP‐2	4.30	0.77	4.02	0.43
Col‐Hep/VEGF			4.48	0.67
Col‐Hep/BMP‐2	5.69	1.08		
Col‐Hep				
Ti/VEGF+BMP‐2	2.56	1.21	4.57	0.40
Ti/VEGF			4.37	1.23
Ti/BMP‐2	2.26	0.77		
Ti Control				

#### 3.1.2 Growth Factor Release

After 3 weeks, the Col‐Hep films with simultaneous loading of rhVEGF165 and rhBMP‐2 released 1.19 μg/cm^2^ (SD 0.30) of rhBMP‐2 and 1.57 μg/cm^2^ of rhVEGF165. The Col‐Hep PEMs loaded with a single growth factor delivered comparable amounts of BMP and VEGF (1.35 μg/cm^2^ (SD 0.16) rhVEGF165 and 1.59 μg/cm^2^ rhBMP‐2, respectively) (Table 2). Uncoated Ti specimens with combined loading with both growth factors released 0.89 μg rhBMP‐2 (SD 0.66) and 0.16 μg of rhVEGF165 (SD 0.05) on average during the observation period of 3 weeks. Release from the bare metal surfaces rhBMP‐2 and rhVEGF165 loading alone produced 0.36 μg/cm^2^ BMP (SD 0.03) and 0.27 μg/cm^2^ VEGF (SD 0.02), respectively.

**TABLE 2:** Cumulative (3 weeks) growth factor release (rhBMP2/rhVEGF_165_).

	rhBMP2 (μg/cm^2^)		rhVEGF_165_ (μg/cm^2^)	
Col‐Hep/VEGF+BMP‐2	1.19	0.30	1.57	0.75
Col‐Hep/VEGF			1.35	0.16
Col‐Hep/BMP‐2	1.59	0.14		
Col‐Hep				
Ti/VEGF+BMP‐2	0.89	0.66	0.16	0.05
Ti/VEGF			0.27	0.02
Ti/BMP‐2	0.36	0.03		
Ti Control				

### 3.2 In Vivo Experiments

All animals recovered well from the operations and were therefore fully included in the evaluation. Postoperative administration of analgesics could be terminated after 5 days. All 96 implants were prepared and evaluated as scheduled.

#### 3.2.1 Histology

##### 3.2.1.1 4 Weeks

**FIGURE 4:**
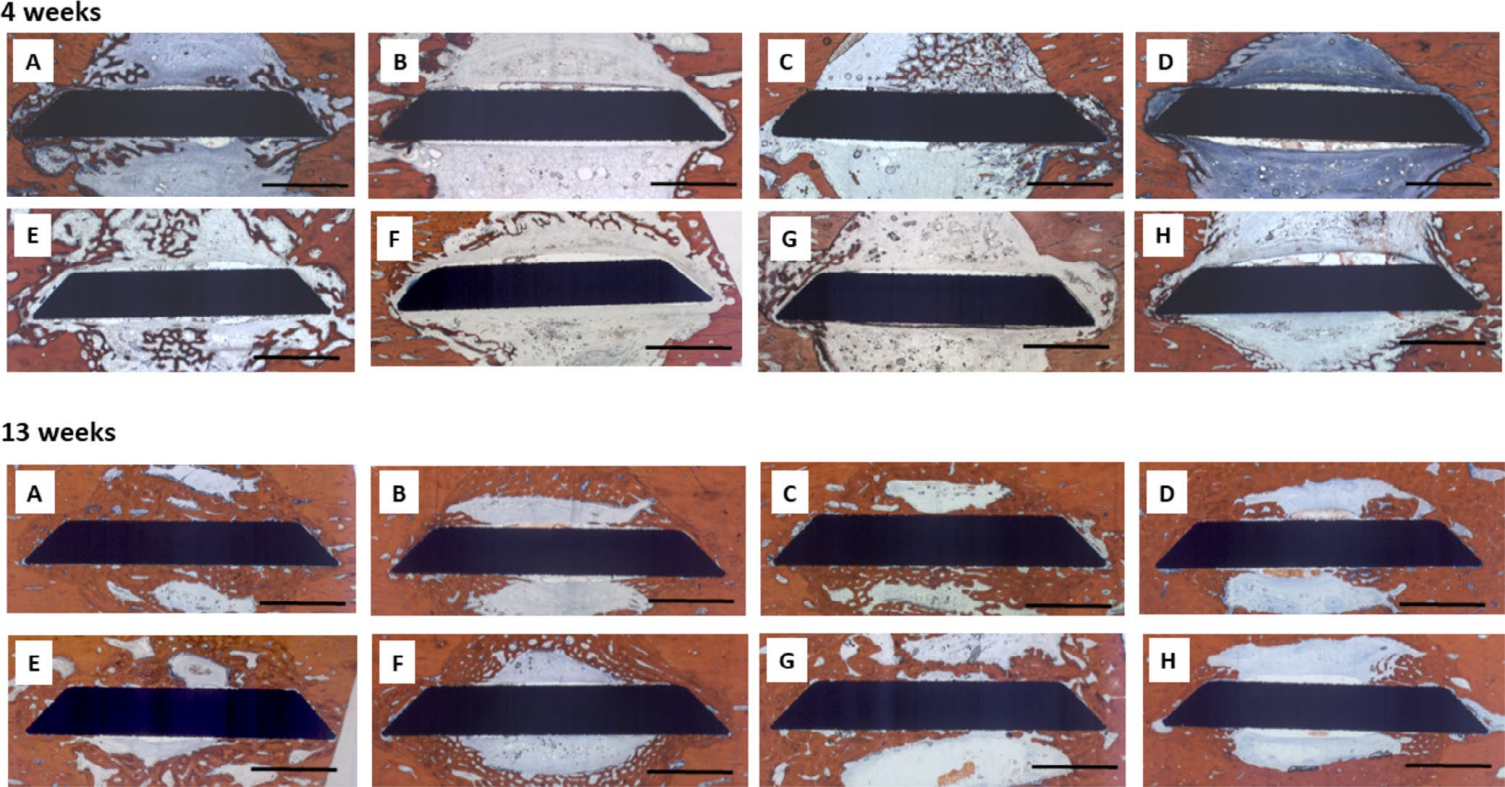
Micrographs of periimplant bone formation: Top Row: 4 weeks, Bottom Row: 13 weeks, Bar: 2000 μm; Top Row: (A) PLL‐HEP1 (COL‐HEP9) multilayer system loaded with rhVEGF165 + rhBMP2, Bone regeneration is visible at the edges of the implant in contact with the defect wall, propagating along the implant surface; (B) PLL‐HEP1 (COL‐HEP9) multilayer system loaded with rhVEGF165: Very little bone formation can be seen at the edges of the implant disc; (C) PLL‐HEP1‐(COL‐HEP9) multilayer system loaded with rhBMP2 showing extensive bone formation on one side of the disc originating from the defect wall; (D) PLL‐HEP1‐(COL‐HEP9 multilayer system) with no appreciable bone regeneration; (E) Uncoated Ti loaded with rhVEGF165 + rhBMP2 exhibiting rather extensive bone formation across the defect; (F) Uncoated Ti loaded with rhVEGF165 showing bone formation only in the periphery of the defect but not at the implant surface; (G) Uncoated Ti loaded with rhBMP2 with little bone formation only on one side of the implant; (H) Uncoated Ti surface unloaded (control): Negligible bone reaction is seen next to the implant. Bottom Row: (A) PLL‐HEP1 (COL‐HEP9) multilayer system loaded with rhVEGF165 + rhBMP2. Considerably matured bone formation is visible with a layer of 1 mm thickness on both sides of the implant; (B) PLL‐HEP1 (COL‐HEP9) multilayer system loaded with rhVEGF165: Rather little bone formation is seen along the implant surface originating from the defect walls; (C) PLL‐HEP1‐(COL‐HEP9) multilayer system loaded with rhBMP2 showing increased bone formation on both implant surfaces; (D) PLL‐HEP1‐(COL‐HEP9 multilayer system) with a clear osteoconductive pattern of bone regeneration; (E) Uncoated Ti loaded with rhVEGF165 + rhBMP2 exhibiting rather extensive bone formation across the defect, but less bone formation at the surface; (F) Uncoated Ti loaded with rhVEGF165 showing rather little bone formation on the central sections of the implant surface but more in the periphery next to the defect wall; (G) Uncoated Ti loaded with rhBMP2 enhanced bone formation across the defect but less bone in contact with the implant surface; (H) Uncoated Ti surface unloaded (control): Bone formation appears to be limited to the implant parts close to the defect wall.

There was only minor bone formation visible after 4 weeks. Osteogenesis appeared to start from the walls of the trephine defect (Figure 4). Thin and immature bone trabeculae filled varying portions of the defect volume, the extent of which was not associated with a specific surface condition of the implant inserted. Bone formation on the implant surface was scarce, with a low level of bone‐to‐implant contact at this observation interval.

**FIGURE 5:**
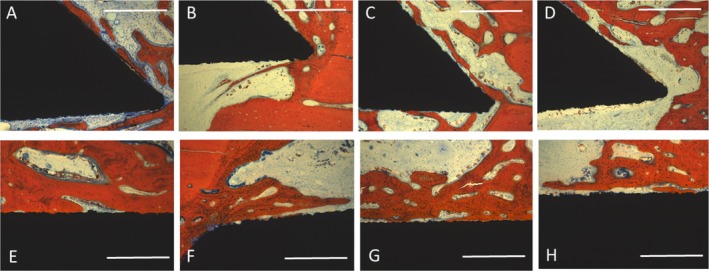
Micrographs of the implant‐bone interface, Bar: 500 μm; (A) (COL‐HEP9) loaded with rhVEGF165 + rhBMP2; 4 weeks, (B) (COL‐HEP9) loaded with rhVEGF165; 4 weeks, (C) (COL‐HEP9) loaded with rhBMP2; 4 weeks, (D) (COL‐HEP9) unloated; 4 weeks; (E) (COL‐HEP9) loaded with rhVEGF165 + rhBMP2; 13 weeks, (F) (COL‐HEP9) loaded with rhVEGF165; 13 weeks, (G) (COL‐HEP9) loaded with rhBMP2; 13 weeks, (H) (COL‐HEP9) unloaded; 13 weeks.

At higher magnification, the multilayer coated implant surfaces loaded with rhBMP2 with and without rhVEGF165 exhibited early bone formation with osteoblast seams on the surface of the edges of the implant discs close to the surrounding bone (Figure 5A,C). Unloaded multilayer coated surfaces and those loaded with rhVEGF only showed less bone contact to surrounding bone with little new bone formation that was not in direct contact with the implant surface (Figure 5B,D).

##### 3.2.1.2 13 Weeks

After 13 weeks, bone formation had matured and filled considerably larger portions of the defect volume with rather large variation between individual animals. There was no appreciable link to specific surface conditions nor growth factor loadings. Osteogenesis adjacent to the implant surface exhibited a clearer pattern of osteoconductive bone formation originating from the defect walls (Figure 4). Implants with Col‐Hep coating and combined loading with rhBMP‐2 and rhVEGF165 showed a more extended coverage of the implant surface with a higher level of bone‐to‐implant contact. At higher magnification, most of the central sections of the multilayer coated surfaces loaded with rhBMP2 and rhVEGF, as well as with rhBMP2 alone, exhibited a continuous bone layer on the surface with varying degrees of maturation (Figure 5E,G). ColHep coated surfaces loaded with rhVEGF165 and those without growth factor loading exhibited bone formation along the surface that was, however, not in direct contact with the implant surface (Figure 5F,H).

### 3.3 Histomorphometry

#### 3.3.1 4 Weeks

##### 3.3.1.1 Bone Formation

**FIGURE 6:**
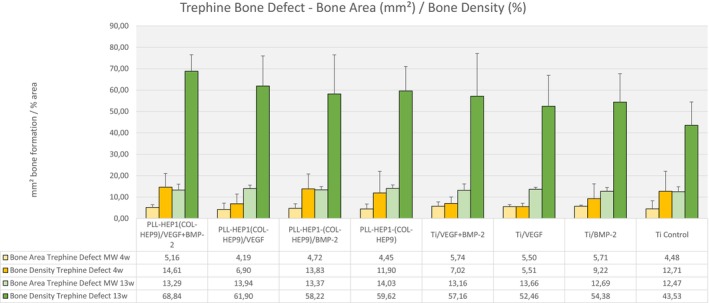
Bone area (mm^2^) and bone density (%) of newly formed bone within the trephine defects. Data are presented as means ± standard deviation (SD) with *n* = 6.

**FIGURE 7:**
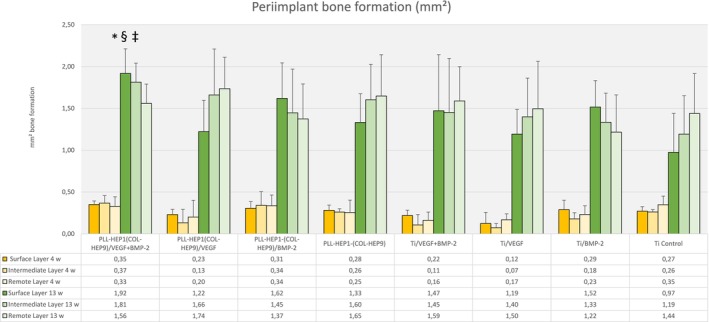
Bone area (mm^2^) of newly formed bone within the periimplant zone (300 μm). Data are presented as means ± standard deviation (SD) with *n* = 6. *Significantly different from Ti control surface (*p* ≤ 0.05), ^§^significantly different from unloaded PEM surface (*p* ≤ 0.05), ^‡^significantly different from PEM surface loaded with rhVEGF165 (*p* ≤ 0.05).

After 4 weeks, the extent of bone formation in the trephine defects varied between 4.19 mm^2^ (Col‐Hep PEM with VEGF loading) and 5.17 mm^2^ (uncoated Ti discs loaded with BMP‐2) with no significant differences between the experimental surface conditions and the controls (*p* = 0.923) (Figure 6). Bone density as well did not differ significantly, exhibiting values between 5.5% (uncoated Ti loaded with VEGF) and 14.6% (Col‐Hep PEM with simultaneous BMP‐2 and VEGF loading) (*p* = 0.159). The degree of bone formation was much lower in the three periimplant zones (immediate, intermediate and remote layer) when compared to the trephine defect, ranging from 0.07 mm^2^ (uncoated Ti loaded with VEGF) to 0.37 mm^2^ (Col‐Hep PEM simultaneous loading with BMP‐2 and VEGF) across all three layers. No significant differences were found at this interval in the individual zones between the individual surface conditions (*p* = 0.464; *p* = 0.112; *p* = 0.379, respectively) (Figure 7).

##### 3.3.1.2 Bone‐Implant Contact

**FIGURE 8:**
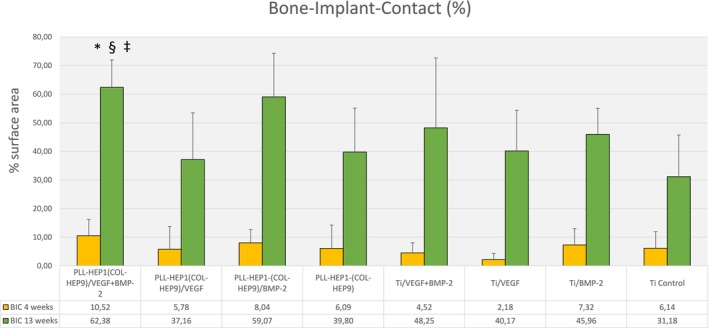
Bone‐Implant‐Contact (%); Data are presented as means ± standard deviation (SD) with *n* = 6; *Significantly different from Ti control surface (*p* ≤ 0.05), ^§^significantly different from unloaded PEM surface (*p* ≤ 0.05), ^‡^significantly different from PEM surface loaded with rhVEGF165 (*p* ≤ 0.05).

After 4 weeks, mean values of bone implant exhibited a rather high degree of variability between the individual surface conditions ranging from 2.61% on average in the group of uncoated Ti implants with VEGF loading to 10.03% in implants with Col‐Hep PEM coating with simultaneous loading with BMP‐2 and VEGF (Figure 8). Despite the relatively large differences between the groups at this time point, there were no significant differences between the different groups of implants with/without PEM coating and with/without growth factor loading due to the high standard deviations (*p* = 0.371).

#### 3.3.2 13 Weeks

##### 3.3.2.1 Bone Formation

Bone formation in the trephine defects as a whole covered an area between 12.7 mm^2^ (uncoated Ti with simultaneous loading with BMP‐2 and VEGF) and 14.03 mm^2^ (unloaded Col‐Hep PEM coating) after 13 weeks with no significant differences between the groups (*p* = 0.923) (Figure 6). Bone density in the trephine defects after 13 weeks was highest in the group of implants with Col‐Hep‐PEM coating and simultaneous loading with BMP‐2 and VEGF (68.8%); the lowest mean value was found in the control group of uncoated and unloaded Ti surfaces (43.5%). Differences between the groups were not significant (*p* = 0.244).

When the three periimplant zones were considered, there were no significant differences between the mean values of newly formed bone area in the intermediate and the remote layer (*p* = 0.173 and *p* = 0.373, respectively). In contrast, the mean area of newly formed bone in the immediate surface layer differed significantly between the individual surface conditions (*p* = 0.043). Implants with Col‐Hep‐PEM coating and simultaneous loading with BMP‐2 and VEGF exhibited the highest mean values (1.92 mm^2^, SD 0.32) and uncoated unloaded Ti controls showed the lowest (0.97 mm^2^, SD 0.51). Pairwise comparison showed that only the PEM coated and simultaneously with BMP and VEGF loaded surfaces induced significantly more bone formation compared to PEM controls and to Ti controls (*p* = 0.028 each) as well as to uncoated Ti loaded with VEGF and with BMP (0.046 each) (Figure 7). Notably, PEM films loaded with VEGF showed significantly less bone formation compared to PEM films with simultaneous loading with BMP and VEGF (*p* = 0.028).

##### 3.3.2.2 Bone‐Implant Contact

After 13 weeks, bone implant contact (BIC) has increased significantly in all implant groups (*p*‐values ranged from 0.002 to 0.026 in the individual groups). The highest mean value has been found in the group of implants with PEM coating and simultaneous growth factor loading of BMP‐2 and VEGF (62.4%, SD 10.5) whereas the lowest was seen in the uncoated unloaded Ti controls (31.2, SD 15.9) (Figure 8). The level of BIC thus mirrored the area of newly formed bone in the immediate surface layer. Pairwise comparison of mean values of BIC between the different surface conditions revealed that implants with PEM coating and simultaneous loading with BMP‐2 and VEGF were the only group that exhibited significantly increased BIC mean values compared to unloaded PEM coating (*p* = 0.046) and uncoated unloaded Ti surfaces (*p* = 0.028). Moreover, the mean BIC rate on PEM films loaded with VEGF only was significantly lower than on the PEMs with simultaneous loading with BMP and VEGF (*p* = 0.046).

### 3.4 Immunofluorescence of CD31 Expression

#### 3.4.1 4 Weeks

**FIGURE 9:**
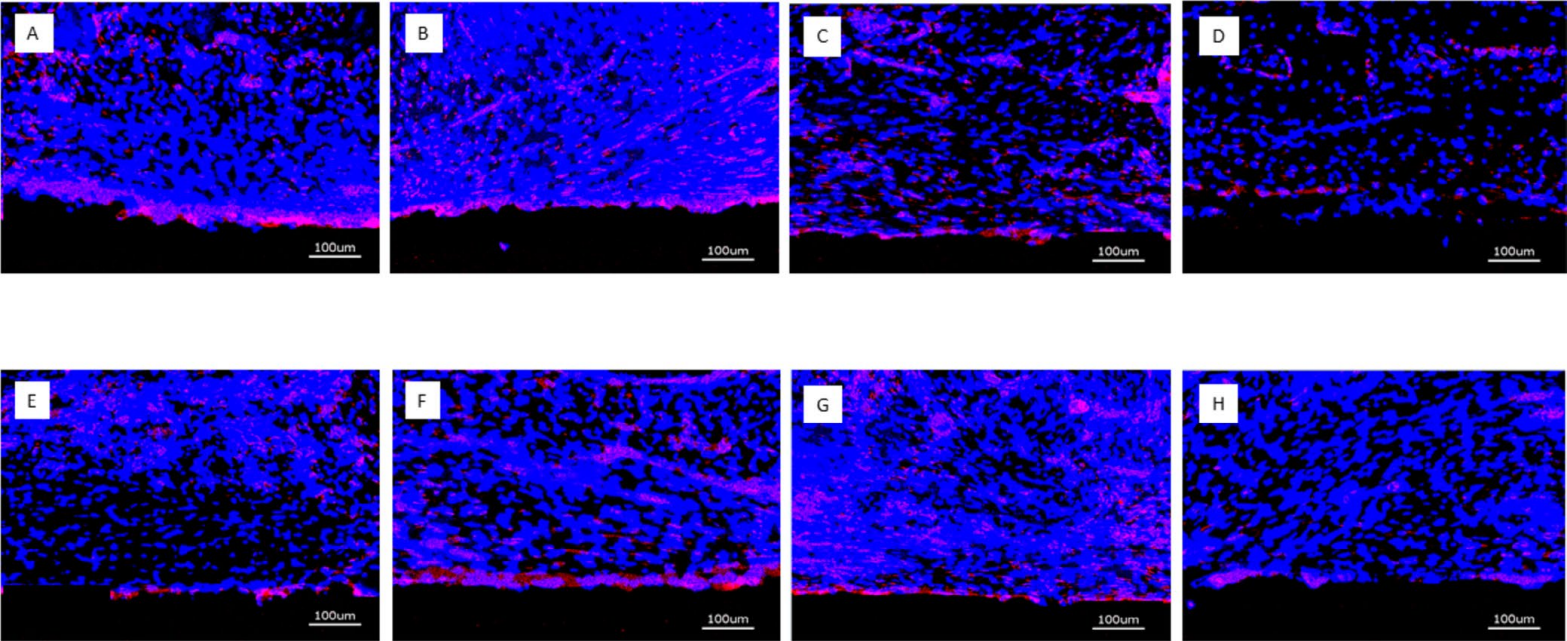
Overview of CD31 positive expression after 4 weeks (bar = 100 μm); (A) PLL‐HEP1 (COL‐HEP9) multilayer system loaded with rhVEGF165 + rhBMP2; (B) PLL‐HEP1 (COL‐HEP9) multilayer system loaded with rhVEGF165; (C) PLL‐HEP1‐(COL‐HEP9) multilayer system loaded with rhBMP2; (D) PLL‐HEP1‐(COL‐HEP9 multilayer system); (E) Uncoated Ti loaded with rhVEGF165 + rhBMP2; (F) Uncoated Ti loaded with rhVEGF165; (G) Uncoated Ti loaded with rhBMP2; (H) Uncoated Ti surface unloaded (control). Red staining indicates the presence of endothelial cells. The PEM coated and uncoated growth factor loaded implants showed a stronger signal immediately next to the implant surface and a diffuse staining for endothelial cells across the overlying tissue, whereas surfaces without growth factor loading did not show this pattern of activity.

Positive staining for CD31 was clearly visible after 4 weeks in a 10–20 μm thick tissue layer in contact with the surface of the implants that had been loaded with BMP and VEGF regardless of combined or single growth factor loading. This held true both for the PEM‐coated and for the uncoated implants. CD31 expression in the soft tissue adjacent to the implant surface showed a diffuse staining pattern across the evaluated regions of interest (Figure 9). The implants without growth factor loading did not exhibit the clear positive staining for CD31 expression next to the implant surface.

#### 3.4.2 13 Weeks

**FIGURE 10:**
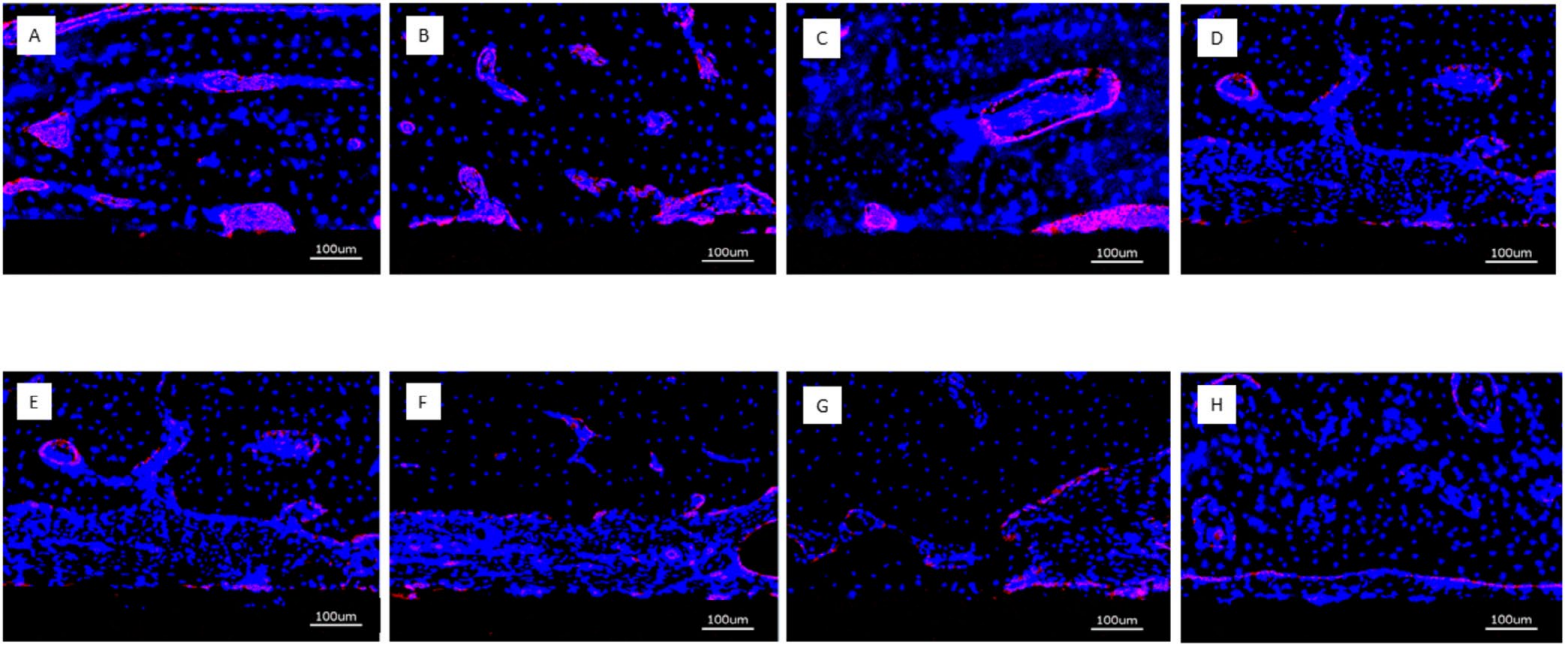
Overview of CD31 positive expression after 13 weeks (bar = 100 μm). (A) PLL‐HEP1 (COL‐HEP9) multilayer system loaded with rhVEGF165 + rhBMP2; (B) PLL‐HEP1 (COL‐HEP9) multilayer system loaded with rhVEGF165; (C) PLL‐HEP1‐(COL‐HEP9) multilayer system loaded with rhBMP2; (D) PLL‐HEP1‐(COL‐HEP9 multilayer system); (E) Uncoated Ti loaded with rhVEGF165 + rhBMP2; (F) Uncoated Ti loaded with rhVEGF165; (G) Uncoated Ti loaded with rhBMP2; (H) Uncoated Ti surface unloaded (control). The accumulation of positive signals next to the implant surface has disappeared and concentrated around blood vessels and bone marrow spaces. The PEM‐coated implants loaded with growth factors showed enhanced signal levels compared to uncoated implants with growth factor loading, indicating a more sustained effect emanating from the PEM‐coated surface.

After 13 weeks, positive staining for CD31 was clearly associated with the perivascular area and the marrow space in the regenerated bone next to the implants with PEM film coating and growth factor loading. No clear difference was visible between the three different states of growth factor loading. Uncoated Ti implants with growth factor loading as well as unloaded Ti controls showed only weak expression of CD31 in the periimplant tissues and had mostly lost the positive staining immediately adjacent to the implant surface (Figure 10).

### 3.5 Histomorphometry of Immunofluorescence of CD31 Expression

#### 3.5.1 4 Weeks

**FIGURE 11:**
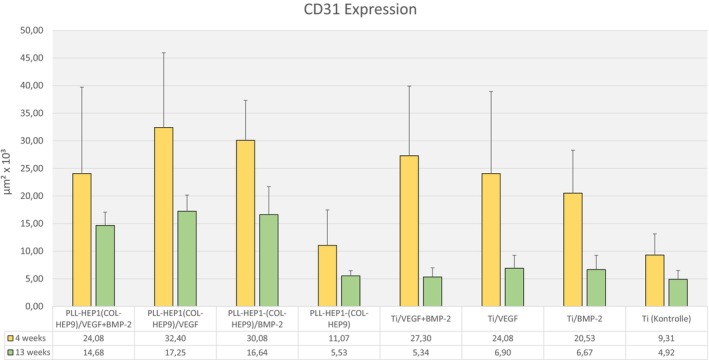
Area of CD31 positive expression (μm^2^/10^3^); Data are presented as means ± standard deviation (SD) with *n* = 6.

The area that had stained positive for CD31 expression varied between the highest mean value of 32.4 × 10^3^ μm^2^ (SD 17.1) in the group of implants with PEM coating and VEGF loading to the lowest mean value of 9.3 × 10^3^ μm^2^ (SD 4.2) in the group of uncoated and unloaded Ti controls (Figure 10). The differences in mean values between all implant groups were highly significant (*p* < 0.001). Pairwise comparison showed that loading of the PEM films with BMP and with VEGF had significantly increased the expression compared to implants coated with the unloaded PEM film (*p* = 0.028 each). When CD31 expression around PEM coated and growth factor loaded implants was compared to uncoated Titanium controls loaded with the corresponding growth factors, there was a significant difference only between BMP‐2 loaded implants (*p* = 0.028) (Table 3). At this observation interval, CD31 expression was also significantly higher in uncoated Ti implants with growth factor loading compared to unloaded Ti discs (Figure 11).

**TABLE 3:** *p*‐values of pairwise comparisons of CD31 expression after 4 weeks (Wilcoxon‐tests).

	PLL‐HEP_1_ (Col‐Hep9)/BMP2 & VEGF	PLL‐HEP_1_ (Col‐Hep9)/VEGF	PLL‐HEP_1_ (Col‐Hep9)/BMP2	PLL‐HEP_1_ (Col‐Hep9)	Ti/VEGF+ BMP‐2	Ti/VEGF	Ti/BMP‐2	Ti control
PLL‐HEP_1_ (Col‐Hep9)/BMP2 & VEGF								
PLL‐HEP_1_ (Col‐Hep9)/VEGF	0.345							
PLL‐HEP_1_ (Col‐Hep9)/BMP2	0.249	0.600						
PLL‐HEP_1_ (Col‐Hep9)	0.116	0.028*	0.028*					
Ti/VEGF+BMP‐2	0.463	0.463	0.345	0.028*				
Ti/VEGF	0.917	0.116	0.249	0.028*				
Ti/BMP‐2	0.917	0.075	0.028*	0.075				
Ti Control	0.046	0.028*	0.028*	0345	0.028*	0.028*	0.028*	

* Significance level *p* < 0.05.

#### 3.5.2 13 Weeks

After 13 weeks, there was a general decrease in expression of CD31 in periimplant tissues compared to the 4‐week interval. These changes were significant for PEM coatings loaded with BMP (*p* = 0.015) and for uncoated Ti controls loaded with VEGF, BMP, and simultaneous loading with both growth factors (*p* = 0.002, *p* = 0.009, and *p* = 0.002, respectively). Areas with positive staining for CD31 expression varied between mean values of 17.3 × 10^3^ μm^3^ (SD 3.2) in the group of PEM‐coated implants with VEGF loading and 4.9 × 10^3^ μm^3^ (SD 1.7) in uncoated and unloaded Ti controls (Figure 10). Again, the differences between all implant groups were highly significant (*p* < 0.001). Differences in the level of CD31 expression between PEM‐coated implants loaded with different growth factor loadings were not significant (Table 4) but the CD31 expression around growth factor‐loaded PEM‐coated implants was significantly higher than implants with unloaded PEM films and uncoated Ti controls loaded with the corresponding growth factors (Table 4). The differences in mean values of CD31 expression between growth factor‐loaded and unloaded Ti controls were not significant anymore (Table 4).

**TABLE 4:** *p*‐values of pairwise comparisons of CD31 expression after 13 weeks (Wilcoxon‐tests).

	PLL‐HEP_1_ (Col‐Hep9)/BMP2 & VEGF	PLL‐HEP_1_ (Col‐Hep9)/VEGF	PLL‐HEP_1_ (Col‐Hep9)/BMP2	PLL‐HEP_1_ (Col‐Hep9)	Ti/VEGF+ BMP‐2	Ti/VEGF	Ti/BMP‐2	Ti control
PLL‐HEP_1_ (Col‐Hep9)/BMP2 & VEGF								
PLL‐HEP_1_ (Col‐Hep9)/VEGF	0.116							
PLL‐HEP_1_ (Col‐Hep9)/BMP2	0.463	0.463						
PLL‐HEP_1_ (Col‐Hep9)	0.028*	0.028*	0.028*					
Ti/VEGF+BMP‐2	0.028*	0.028*	0.028*	0.600				
Ti/VEGF	0.028*	0.028*	0.028*	0.116				
Ti/BMP‐2	0.028*	0.028*	0.028*	0.249				
Ti Control	0.028*	0.028*	0.028*	0.600	0.917	0.249	0.345	

* Significance level *p* < 0.05.

## 4 Discussion

The present study has assessed the effect of dual growth factor release from collagen‐based polyelectrolyte multilayer films (PEMs) on titanium implants on periimplant bone formation and angiogenetic activity. The experimental model used deliberately did not use cylindrical implants with close contact between the implant surface and the surrounding bone in order to provide insights into the reach of the growth factors into a void “periimplant defect” and the regenerating tissue within. The results suggest that the majority of bone regeneration in the trephine cavity occurred independently from the released growth factors and that a significant effect on osteogenesis was only appreciable in a 100 μm tissue layer immediately adjacent to the implant surface. In this tissue layer, only the implants with PEM coating with simultaneous release of BMP‐2 and VEGF165 showed significantly increased bone formation after 13 weeks when compared to implants with unloaded PEM films and unloaded bare titanium controls. This suggests that the osteogenic effect of the growth factors released from the implant surface is limited to a distance of approximately 100 μm in the present experimental model. In order to validate this result, the anatomical situation at the border of the minipig mandible has to be taken into consideration that is characterized by a thick cortical layer and a poorly structured area around the mandibular canal. The experimental model is thus quite challenging both anatomically and biologically as the distance of 1.5 mm between the cortical bone and the implant is unlikely to be filled by spontaneous regeneration after the overlying periosteum has been removed. The results indicate that in a defect situation in mostly cortical bone, little regenerative effect through the growth factor released from the implant surface can be expected, and it is more likely that the growth factor portion retained in the multilayer coating is exerting the main effect, resulting in increased osteoconductive bone formation along the surface from the cavity walls. This may be different when a multilayer‐coated cylindrical implant is inserted into bone of low density with nevertheless close contact between the bone and the implant surface making immediate interaction of the released growth factor with the surrounding bone possible. One may speculate that this may also have implications in loading strategies; however, the current experimental setting does not allow for conclusions in this respect.

It is also interesting to note that the release of sole VEGF from the PEM films was associated with both a significantly lower bone‐to‐implant contact rate and significantly lower bone formation in the 100 μm zone when compared to the PEM coated surfaces releasing BMP and VEGF. This parallels findings from studies looking at different ratios of VEGF and BMP release in bone tissue engineering indicating that a high level of VEGF signaling can be detrimental to the initiation of bone formation by retarding osteogenic differentiation (Moser et al. 2018). A retarding effect of high levels of VEGF on bone formation may be explained by the fact that VEGF has been reported to increase the expression of Runx2 in mesenchymal stem cells (Khojasteh et al. 2016). Runx2 is a marker of osteogenesis, as it is known to promote early stage osteoblastic differentiation. However, for differentiation into mature osteoblasts, Runx2 has to be downregulated (Komori 2010). A prolonged upregulation of the expression of Runx2 through VEGF, thus, may be associated with a retarded process of new bone formation.

The use of polyelectrolyte multilayer coatings with collagen as a cationic partner for Heparin for the immobilization of osteogenic growth factors on the surface of Titanium implants in the present study has shown a stronger osteogenic effect than previous attempts to employ collagen in conjunction with bone growth factors on Titanium surfaces. A number of reports have applied biomimetic coating strategies using self‐assembly of collagen also in combination with glycosaminoglycans (GAGs) such as chondroitin sulfate and hyaluronan, showing a positive effect on cell behavior in vitro and periimplant bone formation in vivo compared to uncoated implant surfaces (Stadlinger et al. 2007; Förster et al. 2017; Norris et al. 2019). The addition of biologically active molecules such as RGD peptides, BMP‐2, or BMP‐4, however, had neither increased periimplant bone formation nor the bone‐implant‐contact rate in these studies (Stadlinger et al. 2007; Schliephake, Aref, et al. 2005; Schliephake et al. 2009). The lack of a positive effect of the integration of osteogenic signaling into the self‐assembled collagen coatings may be due to the fact that the amount of growth factor that is incorporated during the spontaneous assembly approach has been too low to reach an effective level in vivo in periimplant tissues beyond the collagen‐enhanced osteoconductive bone formation on the implant surface (Bierbaum et al. 2005). In contrast, the layer‐by‐layer approach for the construction of PEM films used in the present study is capable of accommodating amounts of rhBMP‐2 of three orders of magnitude higher than previously reported for self‐assembled Collagen‐Proteoglycan films (Bierbaum et al. 2005). In this way, larger amounts of rhBMP2 can be released from the PEM films on the implant surface. Looking at the results of the BMP2 release only, the mean values of bone‐implant‐contact appear to be not much different from those with combined release of BMP and VEGF, but other than the latter, the difference to the controls did not reach statistical significance. This parallels the results of the above‐mentioned studies (Stadlinger et al. 2007; Schliephake et al. 2009) indicating that collagen as such has a strong osteoconductive effect and is not easily surpassed by biologically active factors on the one hand. On the other hand, the outcome corresponds to previously reported results on ridge augmentations in minipigs using the combined release of BMP2 and VEGF165 in that BMP2 required additional release of VEGF165 to achieve a significant osteogenic effect compared to unloaded controls (Kauffmann et al. 2021).

On a clinical level, the use of BMP2 has been limited by a number of problems, one of which is the difficulty of targeted anchoring and retarded release of these molecules to and from a carrier or the metal surface of implants. The adsorptive coating of collagen carriers using solutions of 1.5 mg of BMP2/mL has been associated with a number of adverse events such as osteolysis or soft tissue swelling due to the rapid release of high dosages of growth factors (Burkus et al. 2002; Boyne et al. 2005; Fiorellini et al. 2005) Preclinical experiments with dip coating of titanium implant surfaces using 3 mg/mL BMP have even resulted in partial implant loosening and osteolysis (Leknes et al. 2008) The concentration of BMP and VEGF in the coating solution used in the present study has been 75 μg/mL BMP2 and VEGF165, respectively. This concentration has been identified as the minimum dosage for saturated loading of the multilayers on the implant surface during the in vitro period of evaluation (Behrens, Kauffmann, von Hahn, Giesecke, et al. 2022). It compares well to the BMP concentration used in more recent dip coating approaches of collagen carriers for clinical use in sinus lift procedures (Kim et al. 2015). The amount of BMP incorporated into the multilayer coating in the present study ranged between 4 and 5 μg growth factor/cm^2^ implant surface, of which between 27% and 39% were released during the first 3 weeks, with a substantial portion being retained in the multilayer coating. It is unknown so far whether the early released or the retained portion of growth factor in bioactive coatings accomplishes sustainable effects. The present results suggest that in biologically and geometrically challenging situations, the early release of the reported amounts of BMP is unlikely to lead to increased bone formation or osseointegration, and that biological effects are rather based on the retained amount made available later on during degradation by cells growing on the coated implant surface.

The occurrence of significant osteogenic effects in response to BMP only after 13 weeks also appears to be rather late when compared to frequently used rodent models that have exhibited significant reactions after 4 weeks at latest (Shah et al. 2014) with a levelling off at 12–13 weeks (Schliephake et al. 2008; Lohse et al. 2015; Chang et al. 2007; Hernández et al. 2012; Patel et al. 2008). The late results in the present study, however, parallel reports on the use of bone growth factors in large animal models that almost exclusively have reported significant reactions only after later intervals (Tröltzsch et al. 2017; Kauffmann et al. 2021) reflecting the rather slow reacting bone biology in large animal models compared to rodent models (Pearce et al. 2007).

In contrast to osteogenic signaling, the effect of angiogenic signaling appeared not to be limited to a zone of 100 μm as shown by the immunohistochemical analysis of the expression of CD31. There was a strong positive reaction immediately adjacent to the PEM films that have been growth factor loaded, regardless of BMP, VEGF, or a combination of both, with a diffuse distribution across the region of interest above the implant surface, covering a zone up to approximately 400 μm away from the implant surface after 4 weeks. It is interesting to note that both BMP and VEGF have elicited comparable reactions with CD31 expression that were significantly higher than both unloaded PEMs and Ti controls already after 4 weeks. These results correspond to the fact that CD31 positive endothelial cells constitute a distinct cell population (Type H endothelium) in bone tissue at the distal end of the arterial network that mediates neo‐angiogenesis as part of a specialized tissue environment (Kusumbe et al. 2014). The occurrence of this type of endothelium can be enhanced through HIF1alpha, which upregulates VEGF expression (Ahluwalia and Tarnawski 2012) and may occur during the induction of osteogenesis by the pleiotropic effects of rhBMP‐2 that also include chemotaxis of endothelial cells (Li et al. 2005) and in this way can elicit angiogenic responses independent from VEGF (Wiley and Jin 2011). The results thus suggest that both rhVEGF165 and rhBMP‐2 contribute to the significantly increased endothelial cell population in periimplant tissues. However, other than with osteogenesis, there is no additive effect of rhVEGF165 and rhBMP‐2 on the expression of CD31, as the level of expression has not been enhanced above the effect level of a single growth factor by the combined use of VEGF and BMP. A weakness of the tested CD31 expression is that it indicates endothelial cell identity rather than the formation of new vessels in particular. To prove proper angiogenic activity, an additional marker such as VE‐cadherin or Cadherin‐5 would be suitable, as it is involved in intercellular endothelial contacts and is important for vessel integrity. Future studies should thus evaluate angiogenic activity in a more comprehensive way by displaying not only the level of endothelial cell counts but also a component of intact vessel walls.

A general limitation of the present study is the use of an implant geometry that is different from the clinically used cylindrical or conical shape. While the employed implant design has been chosen to allow for the identification of specific characteristics such as the spatial range of the released growth factors and osteoconductive vs. osteoinductive bone formation under the influence of the released growth factors, immediate translation into the clinical situation is difficult. Future in vivo experiments should use implant shapes of commercially available implants in an osteoporotic large animal model to assess the efficacy of the developed multilayer coating in conjunction with bone growth factors in an experimental setting that is closer to the clinical situation.

In conclusion, the present study has shown that collagen‐heparin based polyelectrolyte multilayer films on micro‐rough titanium surfaces can accommodate rhBMP‐2 and rhVEGF165 in clinically relevant amounts and induce a significant increase in both peri‐implant bone formation and bone‐implant contact when both growth factors are released together. In the present model, the appreciable range of osteogenic activity appears to be limited to a 100 μm distance away from the implant surface beyond which it is overruled by the osteogenetic activity of the regenerating pristine bone. Angiogenetic activity, as detected by the expression of CD31, is significantly increased by both rhBMP‐2 and rhVEGF165 at a comparable level without an additive effect of a combined release. The enhancement of CD31 expression by the released growth factors does not appear to be limited to a range of 100 μm.

## Author Contributions

**Philipp Kauffmann:** conceptualization, methodology, data curation, writing – review and editing. **Susanne Wolfer:** investigation, data curation, writing – review and editing. **Christina Behrens:** conceptualization, methodology, investigation, visualization. **Pauline Schlosser:** software, investigation, data curation, writing – review and editing. **Christian Dullin:** software, writing – review and editing. **Uwe Schirmer:** investigation, visualization, writing – review and editing. **Klaus Liefeith:** conceptualization, methodology, supervision, project administration, resources, writing – review and editing. **Henning Schliephake:** conceptualization, methodology, validation, supervision, funding acquisition, resources, writing – original draft, writing – review and editing, formal analysis.

## Conflicts of Interest

The authors declare no conflicts of interest.

## Data Availability

Data supporting the reported results are available as Excel files on request at schliephake.henning@med.uni-goettingen.de.

## References

[clr70045-bib-0001] Ahluwalia, A. , and A. S. Tarnawski . 2012. “Critical Role of Hypoxia Sensor—HIF‐1α in VEGF Gene Activation. Implications for Angiogenesis and Tissue Injury Healing.” Current Medicinal Chemistry 19, no. 1: 90–97. 10.2174/092986712803413944. 22300081

[clr70045-bib-0002] Ao, H. , Y. Xie , H. Tan , et al. 2013. “Fabrication and In Vitro Evaluation of Stable Collagen/Hyaluronic Acid Biomimetic Multilayer on Titanium Coatings.” Journal of the Royal Society Interface 10: 20130070. 10.1098/rsif.2013.0070. 23635490 PMC3673146

[clr70045-bib-0003] Bayer, E. A. , R. Gottardi , M. V. Fedorchak , and S. R. Little . 2015. “The Scope and Sequence of Growth Factor Delivery for Vascularized Bone Tissue Regeneration.” Journal of Controlled Release 219: 129–140. 10.1016/j.jconrel.2015.08.004. 26264834

[clr70045-bib-0004] Behrens, C. , P. Kauffmann , N. von Hahn , et al. 2022. “Development of a System of Heparin Multilayers on Titanium Surfaces for Dual Growth Factor Release.” Journal of Biomedical Materials Research. Part A 110, no. 9: 1599–1615. 10.1002/jbm.a.37411. 35593380

[clr70045-bib-0005] Behrens, C. , P. Kauffmann , N. von Hahn , U. Schirmer , K. Liefeith , and H. Schliephake . 2022. “Collagen‐Based Osteogenic Nanocoating of Microrough Titanium Surfaces.” International Journal of Molecular Sciences 23, no. 14: 7803. 10.3390/ijms23147803. 35887152 PMC9317921

[clr70045-bib-0006] Bierbaum, S. , S. Roessler , T. Douglas , et al. 2005. “Collagen Matrix Composition and Structure—Influence on Binding, Release and Activity of TGF‐b1, BMP‐2 and BMP‐4.” *Proceedings of the 18th European Conference on Biomaterials*. Sorrento, Italy, T43–740.

[clr70045-bib-0007] Bouyer, M. , R. Guillot , J. Lavaud , et al. 2016. “Surface Delivery of Tunable Doses of BMP‐2 From an Adaptable Polymeric Scaffold Induces Volumetric Bone Regeneration.” Biomaterials 104: 168–181. 10.1016/j.biomaterials.2016.06.001. 27454063 PMC5937675

[clr70045-bib-0008] Boyne, P. J. , L. C. Lilly , R. E. Marx , et al. 2005. “De Novo Bone Induction by Recombinant Human Bone Morphogenetic Protein‐2 (rhBMP‐2) in Maxillary Sinus Floor Augmentation.” Journal of Oral and Maxillofacial Surgery 63, no. 12: 1693–1707. 10.1016/j.joms.2005.08.018. 16297689

[clr70045-bib-0009] Brito Barrera, Y. A. , G. Hause , M. Menzel , et al. 2020. “Engineering Osteogenic Microenvironments by Combination of Multilayers From Collagen Type I and Chondroitin Sulfate With Novel Cationic Liposomes.” Materials Today Bio 7: 100071. 10.1016/j.mtbio.2020.100071. PMC7476072 32924006

[clr70045-bib-0010] Burkus, J. K. , E. E. Transfeldt , S. H. Kitchel , R. G. Watkins , and R. A. Balderston . 2002. “Clinical and Radiographic Outcomes of Anterior Lumbar Interbody Fusion Using Recombinant Human Bone Morphogenetic Protein‐2.” Spine 27, no. 21: 2396–2408. 10.1097/00007632-200211010-00015. 12438990

[clr70045-bib-0011] Chang, P. C. , B. Y. Liu , C. M. Liu , et al. 2007. “Bone Tissue Engineering With Novel rhBMP2‐PLLA Composite Scaffolds.” Journal of Biomedical Materials Research. Part A 81, no. 4: 771–780. 10.1002/jbm.a.31031. 17226806

[clr70045-bib-0012] Damanik, F. F. R. , M. Brunelli , L. Pastorino , et al. 2019. “Sustained Delivery of Growth Factors With High Loading Efficiency in a Layer by Layer Assembly.” Biomaterials Science 8, no. 1: 174–188. 10.1039/c9bm00979e. 31713550

[clr70045-bib-0013] Donath, K. , and G. Breuner . 1982. “A Method for the Study of Undecalcified Bones and Teeth With Attached Soft Tissues. The Säge‐Schliff (Sawing and Grinding) Technique.” Journal of Oral Pathology 11, no. 4: 318–326. 10.1111/j.1600-0714.1982.tb00172.x. 6809919

[clr70045-bib-0014] Ferreira, A. M. , P. Gentile , S. Toumpaniari , G. Ciardelli , and M. A. Birch . 2016. “Impact of Collagen/Heparin Multilayers for Regulating Bone Cellular Functions.” ACS Applied Materials & Interfaces 8, no. 44: 29923–29932. 10.1021/acsami.6b09241. 27762547

[clr70045-bib-0015] Fiorellini, J. P. , T. H. Howell , D. Cochran , et al. 2005. “Randomized Study Evaluating Recombinant Human Bone Morphogenetic Protein‐2 for Extraction Socket Augmentation.” Journal of Periodontology 76, no. 4: 605–613. 10.1902/jop.2005.76.4.605. 15857102

[clr70045-bib-0016] Förster, Y. , R. Bernhardt , V. Hintze , et al. 2017. “Collagen/Glycosaminoglycan Coatings Enhance New Bone Formation in a Critical Size Bone Defect—A Pilot Study in Rats.” Materials Science & Engineering, C: Materials for Biological Applications 71: 84–92. 10.1016/j.msec.2016.09.071. 27987780

[clr70045-bib-0017] Gilde, F. , L. Fourel , R. Guillot , et al. 2016. “Stiffness‐Dependent Cellular Internalization of Matrix‐Bound BMP‐2 and Its Relation to Smad and Non‐Smad Signalling.” Acta Biomaterialia 46: 55–67. 10.1016/j.actbio.2016.09.014. 27633320 PMC5113753

[clr70045-bib-0018] Gronowicz, G. , E. Jacobs , T. Peng , L. Zhu , M. Hurley , and L. T. Kuhn . 2017. “Calvarial Bone Regeneration Is Enhanced by Sequential Delivery of FGF‐2 and BMP‐2 From Layer‐by‐Layer Coatings With a Biomimetic Calcium Phosphate Barrier Layer.” Tissue Engineering, Part A 23, no. 23–24: 1490–1501. 10.1089/ten.TEA.2017.0111. 28946792 PMC5729881

[clr70045-bib-0019] Guillot, R. , F. Gilde , P. Becquart , et al. 2013. “The Stability of BMP Loaded Polyelectrolyte Multilayer Coatings on Titanium.” Biomaterials 34: 5737–5746. 10.1016/j.biomaterials.2013.03.067. 23642539 PMC4119881

[clr70045-bib-0020] Hernández, A. , R. Reyes , E. Sánchez , M. Rodríguez‐Évora , A. Delgado , and C. Evora . 2012. “In Vivo Osteogenic Response to Different Ratios of BMP‐2 and VEGF Released From a Biodegradable Porous System.” Journal of Biomedical Materials Research. Part A 100, no. 9: 2382–2391. 10.1002/jbm.a.34183. 22528545

[clr70045-bib-0021] Huang, J. , J. Lu , Z. Liu , et al. 2022. “Covalent Immobilization of VEGF on Allogeneic Bone Through Polydopamine Coating to Improve Bone Regeneration.” Frontiers in Bioengineering and Biotechnology 10: 1003677. 10.3389/fbioe.2022.1003677. 36312529 PMC9597090

[clr70045-bib-0022] Hwang, J. H. , U. Han , M. Yang , et al. 2019. “Artificial Cellular Nano‐Environment Composed of Collagen‐Based Nanofilm Promotes Osteogenic Differentiation of Mesenchymal Stem Cells.” Acta Biomaterialia 86: 247–256. 10.1016/j.actbio.2018.12.044. 30594632

[clr70045-bib-0023] Hynes, R. O. 2002. “Integrins: Bidirectional, Allosteric Signaling Machines.” Cell 110, no. 6: 673–687. 10.1016/s0092-8674(02)00971-6. 12297042

[clr70045-bib-0024] Ishihara, M. , S. Nakamura , Y. Sato , et al. 2019. “Heparinoid Complex‐Based Heparin‐Binding Cytokines and Cell Delivery Carriers.” Molecules 24, no. 24: 4630. 10.3390/molecules24244630. 31861225 PMC6943580

[clr70045-bib-0025] Jacobs, E. E. , G. Gronowicz , M. M. Hurley , and L. T. Kuhn . 2017. “Biomimetic Calcium Phosphate/Polyelectrolyte Multilayer Coatings for Sequential Delivery of Multiple Biological Factors.” Journal of Biomedical Materials Research. Part A 105, no. 5: 1500–1509. 10.1002/jbm.a.35985. 28002652 PMC5378589

[clr70045-bib-0026] Kauffmann, P. , D. Raschke , M. Tröltzsch , P. Santander , P. Brockmeyer , and H. Schliephake . 2021. “The Use of rhBMP2 for Augmentation of Established Horizontal/Vertical Defects May Require Additional Use of rhVEGF to Achieve Significant Bone Regeneration: An In Vivo Experimental Study.” Clinical Oral Implants Research 32, no. 10: 1228–1240. 10.1111/clr.13820. 34352150

[clr70045-bib-0027] Kauffmann, P. , S. Wolfer , C. Behrens , et al. 2025. “Effect of Sequential vs. Simultaneous Dual Growth Factor Release From Structured Heparin‐Polyelectrolyte‐Multilayer Coatings on Periimplant Bone Formation and Angiogenesis in Pig Mandibles.” Journal of Functional Biomaterials 16, no. 2: 67. 10.3390/jfb16020067. 39997601 PMC11857039

[clr70045-bib-0028] Keller, J. C. , M. Stewart , M. Roehm , and G. B. Schneider . 2004. “Osteoporosis‐Like Bone Conditions Affect Osseointegration of Implants.” International Journal of Oral & Maxillofacial Implants 19: 687–694. 15508984

[clr70045-bib-0029] Khojasteh, A. , F. Fahimipour , M. B. Eslaminejad , et al. 2016. “Development of PGLA‐Coated β‐TCP Scaffolds Containing VEGF for Bone Tissue Engineering.” Materials Science & Engineering, C: Materials for Biological Applications 69: 780–788. 27612772 10.1016/j.msec.2016.07.011

[clr70045-bib-0030] Kilkenny, C. , W. J. Browne , I. C. Cuthill , M. Emerson , and D. G. Altman . 2012. “Improving Bioscience Research Reporting: The ARRIVE Guidelines for Reporting Animal Research.” Osteoarthritis and Cartilage 20, no. 4: 256–260. 10.1016/j.joca.2012.02.010. 22424462

[clr70045-bib-0031] Kim, M. S. , J. S. Lee , H. K. Shin , J. S. Kim , J. H. Yun , and K. S. Cho . 2015. “Prospective Randomized, Controlled Trial of Sinus Grafting Using Escherichia‐Coli‐Produced rhBMP‐2 With a Biphasic Calcium Phosphate Carrier Compared to Deproteinized Bovine Bone.” Clinical Oral Implants Research 26, no. 12: 1361–1368. 10.1111/clr.12471. 25186180

[clr70045-bib-0032] Komori, T. 2010. “Regulation of Bone Development and Extracellular Matrix Protein Genes by RUNX2.” Cell and Tissue Research 339: 189–195. 19649655 10.1007/s00441-009-0832-8

[clr70045-bib-0033] Kunrath, M. F. , C. Garaicoa‐Pazmino , P. M. Giraldo‐Osorno , et al. 2024. “Implant Surface Modifications and Their Impact on Osseointegration and Peri‐Implant Diseases Through Epigenetic Changes: A Scoping Review.” Journal of Periodontal Research 59, no. 6: 1095–1114. 10.1111/jre.13273. 38747072 PMC11626700

[clr70045-bib-0034] Kusumbe, A. P. , S. K. Ramasamy , and R. H. Adams . 2014. “Coupling of Angiogenesis and Osteogenesis by a Specific Vessel Subtype in Bone.” Nature 507, no. 7492: 323–328. 10.1038/nature13145. 24646994 PMC4943525

[clr70045-bib-0035] Leknes, K. N. , J. Yang , M. Qahash , G. Polimeni , C. Susin , and U. M. Wikesjö . 2008. “Alveolar Ridge Augmentation Using Implants Coated With Recombinant Human Bone Morphogenetic Protein‐2: Radiographic Observations.” Clinical Oral Implants Research 19, no. 10: 1027–1033. 10.1111/j.1600-0501.2008.01567.x. 18828819

[clr70045-bib-0036] Lemos, C. A. A. , A. S. de Oliveira , D. S. Faé , et al. 2023. “Do Dental Implants Placed in Patients With Osteoporosis Have Higher Risks of Failure and Marginal Bone Loss Compared to Those in Healthy Patients? A Systematic Review With Meta‐Analysis.” Clinical Oral Investigations 27, no. 6: 2483–2493. 10.1007/s00784-023-05005-2. 37043030

[clr70045-bib-0037] Li, G. , Y. Cui , L. McIlmurray , W. E. Allen , and H. Wang . 2005. “rhBMP‐2, rhVEGF(165), rhPTN and Thrombin‐Related Peptide, TP508 Induce Chemotaxis of Human Osteoblasts and Microvascular Endothelial Cells.” Journal of Orthopaedic Research 23, no. 3: 680–685. 10.1016/j.orthres.2004.12.005. 15885491

[clr70045-bib-0038] Liu, X. , W. C. Liu , H. Y. Wang , et al. 2021. “Polyelectrolyte Multilayer Composite Coating on 316 L Stainless Steel for Controlled Release of Dual Growth Factors Accelerating Restoration of Bone Defects.” Materials Science & Engineering, C: Materials for Biological Applications 126: 112187. 10.1016/j.msec.2021.112187. 34082986

[clr70045-bib-0039] Lohse, N. , N. Moser , S. Backhaus , T. Annen , M. Epple , and H. Schliephake . 2015. “Continuous Delivery of rhBMP2 and rhVEGF165 at a Certain Ratio Enhances Bone Formation in Mandibular Defects Over the Delivery of rhBMP2 Alone—An Experimental Study in Rats.” Journal of Controlled Release 220: 201–209. 10.1016/j.jconrel.2015.10.032. 26485046

[clr70045-bib-0040] Lu, Y. T. , P. T. Hung , K. Zeng , et al. 2023. “Sustained Growth Factor Delivery From Bioactive PNIPAM‐Grafted‐Chitosan/Heparin Multilayers as a Tool to Promote Growth and Migration of Cells.” Biomaterials Advances 154: 213589. 10.1016/j.bioadv.2023.213589. 37598438

[clr70045-bib-0041] Ludolph, J. , H. Rothe , U. Schirmer , et al. 2022. “Tailored Polyelectrolyte Multilayer Systems by Variation of Polyelectrolyte Composition and EDC/NHS Cross‐Linking: Controlled Drug Release vs. Drug Reservoir Capabilities and Cellular Response for Improved Osseointegration.” Polymers 14, no. 20: 4315. 10.3390/polym14204315. 36297892 PMC9609345

[clr70045-bib-0042] Moser, N. , J. Goldstein , P. Kauffmann , M. Epple , and H. Schliephake . 2018. “Experimental Variation of the Level and the Ratio of Angiogenic and Osteogenic Signaling Affects the Spatiotemporal Expression of Bone‐Specific Markers and Organization of Bone Formation in Ectopic Sites.” Clinical Oral Investigations 22, no. 3: 1223–1234. 10.1007/s00784-017-2202-3. 28936783

[clr70045-bib-0043] Norris, K. , O. I. Mishukova , A. Zykwinska , et al. 2019. “Marine Polysaccharide‐Collagen Coatings on Ti6Al4V Alloy Formed by Self‐Assembly.” Micromachines 10: 68–74. 10.3390/mi10010068. 30669427 PMC6356479

[clr70045-bib-0044] Patel, Z. S. , S. Young , Y. Tabata , J. A. Jansen , M. E. Wong , and A. G. Mikos . 2008. “Dual Delivery of an Angiogenic and an Osteogenic Growth Factor for Bone Regeneration in a Critical Size Defect Model.” Bone 43, no. 5: 931–940. 10.1016/j.bone.2008.06.019. 18675385 PMC3014108

[clr70045-bib-0045] Pearce, A. I. , R. G. Richards , S. Milz , E. Schneider , and S. G. Pearce . 2007. “Animal Models for Implant Biomaterial Research in Bone: A Review.” European Cells and Materials 13: 1–10. 17334975 10.22203/ecm.v013a01

[clr70045-bib-0046] Ramazanoglu, M. , R. Lutz , C. Ergun , C. von Wilmowsky , E. Nkenke , and K. A. Schlegel . 2011. “The Effect of Combined Delivery of Recombinant Human Bone Morphogenetic Protein‐2 and Recombinant Human Vascular Endothelial Growth Factor 165 From Biomimetic Calcium‐Phosphate‐Coated Implants on Osseointegration.” Clinical Oral Implants Research 22, no. 12: 1433–1439. 10.1111/j.1600-0501.2010.02133.x. 21418332

[clr70045-bib-0047] Ramazanoglu, M. , R. Lutz , P. Rusche , et al. 2013. “Bone Response to Biomimetic Implants Delivering BMP‐2 and VEGF: An Immunohistochemical Study.” Journal of Craniofacial Surgery 41, no. 8: 826–835. 10.1016/j.jcms.2013.01.037. 23434516

[clr70045-bib-0048] Sarvaiya, B. B. , S. Kumar , M. S. H. Pathan , S. Patel , V. Gupta , and M. Haque . 2025. “The Impact of Implant Surface Modifications on the Osseointegration Process: An Overview.” Cureus 17, no. 4: e81576. 10.7759/cureus.81576. 40177230 PMC11961139

[clr70045-bib-0049] Scharnweber, D. , F. Schlottig , S. Oswald , K. Becker , and H. Worch . 2010. “How Is Wettability of Titanium Surfaces Influenced by Their Preparation and Storage Conditions?” Journal of Materials Science: Materials in Medicine 21: 525e–532e. 19851840 10.1007/s10856-009-3908-9

[clr70045-bib-0050] Schiegnitz, E. , K. Reinicke , K. Sagheb , J. König , B. Al‐Nawas , and K. A. Grötz . 2022. “Dental Implants in Patients With Head and Neck Cancer‐A Systematic Review and Meta‐ Analysis of the Influence of Radiotherapy on Implant Survival.” Clinical Oral Implants Research 33, no. 10: 967–999. 10.1111/clr.13976. 35841367

[clr70045-bib-0052] Schliephake, H. , A. Aref , D. Scharnweber , S. Bierbaum , S. Roessler , and A. Sewing . 2005. “Effect of Immobilized Bone Morphogenic Protein 2 Coating of Titanium Implants on Periimplant Bone Formation.” Clinical Oral Implants Research 16: 563–569. 16164462 10.1111/j.1600-0501.2005.01143.x

[clr70045-bib-0053] Schliephake, H. , A. Aref , D. Scharnweber , S. Bierbaum , and A. Sewing . 2009. “Effect of Modifications of Dual Acid‐Etched Implant Surfaces on Peri‐Implant Bone Formation. Part I: Organic Coatings.” Clinical Oral Implants Research 20: 31–37. 10.1111/j.1600-0501.2008.01603.x. 19126105

[clr70045-bib-0054] Schliephake, H. , C. Bötel , A. Förster , B. Schwenzer , J. Reichert , and D. Scharnweber . 2012. “Effect of Oligonucleotide Mediated Immobilization of Bone Morphogenic Proteins on Titanium Surfaces—An In Vitro Study.” Biomaterials 33: 1315–1322. 22082620 10.1016/j.biomaterials.2011.10.027

[clr70045-bib-0051] Schliephake, H. , and K. Liefeith . 2022. “Tailored Polyelectrolyte Multilayer Systems by Variation of Polyelectrolyte Composition and EDC/NHS Cross‐Linking: Physicochemical Characterization and In Vitro Evaluation.” Nanomaterials 12, no. 12: 2054. 10.3390/nano12122054. 35745395 PMC9228333

[clr70045-bib-0055] Schliephake, H. , D. Scharnweber , M. Dard , A. Sewing , A. Aref , and S. Roessler . 2005. “Functionalization of Dental Implant Surfaces Using Adhesion Molecules.” Journal of Biomedical Materials Research. Part B, Applied Biomaterials 73, no. 1: 88–96. 10.1002/jbm.b.30183. 15786448

[clr70045-bib-0056] Schliephake, H. , H. A. Weich , C. Dullin , R. Gruber , and S. Frahse . 2008. “Mandibular Bone Repair by Implantation of rhBMP‐2 in a Slow Release Carrier of Polylactic Acid—An Experimental Study in Rats.” Biomaterials 29, no. 1: 103–110. 10.1016/j.biomaterials.2007.09.019. 17936352

[clr70045-bib-0057] Shah, N. J. , M. N. Hyder , M. A. Quadir , et al. 2014. “Adaptive Growth Factor Delivery From a Polyelectrolyte Coating Promotes Synergistic Bone Tissue Repair and Reconstruction.” Proceedings of the National Academy of Sciences of the United States of America 111, no. 35: 12847–12852. 10.1073/pnas.1408035111. 25136093 PMC4156697

[clr70045-bib-0058] Shi, Z. , K. G. Neoh , E. T. Kang , C. K. Poh , and W. Wang . 2009. “Surface Functionalization of Titanium With Carboxymethyl Chitosan and Immobilized Bone Morphogenetic Protein‐2 for Enhanced Osseointegration.” Biomacromolecules 10, no. 6: 1603–1611. 10.1021/bm900203w. 19391583

[clr70045-bib-0059] Souza, J. C. M. , M. B. Sordi , M. Kanazawa , et al. 2019. “Nano‐Scale Modification of Titanium Implant Surfaces to Enhance Osseointegration.” Acta Biomaterialia 94: 112–131. 10.1016/j.actbio.2019.05.045. 31128320

[clr70045-bib-0060] Stadlinger, B. , E. Pilling , M. Hulhe , et al. 2007. “Influence of Extracellular Matrix Coatings on Implant Stability and Osseointegration: An Animal Study.” Journal of Biomedical Materials Research 83: 222–231. 17318830 10.1002/jbm.b.30787

[clr70045-bib-0061] Temmerman, A. , L. Rasmusson , A. Kübler , A. Thor , J. Merheb , and M. Quirynen . 2019. “A Prospective, Controlled, Multicenter Study to Evaluate the Clinical Outcome of Implant Treatment in Women With Osteoporosis/Osteopenia: 5‐Year Results.” Journal of Dental Research 98, no. 1: 84–90. 10.1177/0022034518798804. 30205020

[clr70045-bib-0062] Tröltzsch, M. , A. Klenke , P. Santander , et al. 2017. “Repair of Large Saddle Defects of the Mandibular Ridge Using Dual Growth Factor Release—An Experimental Pilot Study in Minipigs.” Journal of Clinical Periodontology 44, no. 8: 854–863. 10.1111/jcpe.12739. 28453232

[clr70045-bib-0063] Wigmosta, T. B. , K. C. Popat , and M. J. Kipper . 2021. “Bone Morphogenetic Protein‐2 Delivery From Polyelectrolyte Multilayers Enhances Osteogenic Activity on Nanostructured Titania.” Journal of Biomedical Materials Research. Part A 109, no. 7: 1173–1182. 10.1002/jbm.a.37109. 32985077

[clr70045-bib-0064] Wiley, D. M. , and S. W. Jin . 2011. “Bone Morphogenetic Protein Functions as a Context‐Dependent Angiogenic Cue in Vertebrates.” Seminars in Cell & Developmental Biology 22, no. 9: 1012–1018. 10.1016/j.semcdb.2011.10.005. 22008724 PMC3548572

[clr70045-bib-0065] Zhang, B. J. , Z. W. Han , K. Duan , Y. D. Mu , and J. Weng . 2018. “Multilayered Pore‐Closed PLGA Microsphere Delivering OGP and BMP‐2 in Sequential Release Patterns for the Facilitation of BMSCs Osteogenic Differentiation.” Journal of Biomedical Materials Research. Part A 106, no. 1: 95–105. 10.1002/jbm.a.36210. 28884494

[clr70045-bib-0066] Zhang, Y. , C. Zhou , Q. Xie , et al. 2025. “Dual Release Scaffolds as a Promising Strategy for Enhancing Bone Regeneration: An Updated Review.” Nanomedicine (London) 20, no. 4: 371–388. 10.1080/17435889.2025.2457317. PMC11812394 39891431

